# Higher density of CD4+ T cell infiltration predicts severe renal lesions and renal function decline in patients with diabetic nephropathy

**DOI:** 10.3389/fimmu.2024.1474377

**Published:** 2024-11-25

**Authors:** Qianqian Han, Huan Xu, Lin Li, Song Lei, Ziyao Li, Lijun Zhao, Fang Liu

**Affiliations:** ^1^ Department of Pathology, West China Hospital of Sichuan University, Chengdu, China; ^2^ Department of General Practice Ward/International Medical Center Ward, General Practice Medical Center, West China Hospital, Sichuan University, Chengdu, China; ^3^ Department of Nephrology, Laboratory of Diabetic Kidney Disease, Kidney Research Institute, West China Hospital of Sichuan University, Chengdu, China

**Keywords:** diabetic nephropathy, CD4+ T cells, tubular atrophy, interstitial fibrosis, clinicopathological analysis

## Abstract

**Background:**

More evidence have shown that the combination of immune and inflammatory mechanism was critical in diabetic nephropathy (DN). However, the relationship between CD4+ T cells and the development of DN is still unclear. Therefore, this study will focus on this issue from the perspective of clinicopathology.

**Methods:**

From September 2019 to December 2022, a total of 112 adult patients with DN were enrolled in the study. According to the density of CD4+ T cell infiltration based on immunostaining, the patients were divided into high-CD4 group (56 patients) and low-CD4 group (56 patients). Another 25 diabetic patients with minimal change disease (non-diabetic nephropathy, NDN) was reviewed as control group in clinical and molecular analysis. The clinical parameters, morphological features, and molecular characteristics were compared. The predictive value of CD4+ T cells for DN prognosis was also investigated.

**Results:**

DN patients in the high-CD4 group suffered from higher proteinuria and lower estimated glomerular filtration rate (eGFR) level than those in the low-CD4 group and NDN patients. Renal biopsy in the high-CD4 group presented with more severe glomerular lesions, higher density of interstitial inflammation, and more severe tubular atrophy/interstitial fibrosis than in the low-CD4 group. Multivariate logistic analysis indicated that the density of CD4+ T cell infiltration could independently predict the severity of tubular atrophy/interstitial fibrosis. In addition, more severe mitochondrial damage of renal tubular epithelial cells and a more obvious expression of Bcl6, IL-6, STAT3, and TGFβ1 were observed in DN patients of the high-CD4 group, indicating the possible mechanism of CD4+ T cells involving the progression of DN. Multivariate Cox regression analysis revealed that a higher intensity of interstitial CD4+ T cell deposition remained as an independent predictor of the double endpoint with doubling of baseline serum creatinine or end-stage renal disease.

**Conclusion:**

The high density of CD4+ T cell infiltration was associated with renal function decline and severity of renal lesions and predicted poor renal survival for DN patients.

## Introduction

Diabetic nephropathy (DN) is a chronic kidney disease caused by diabetic mellitus (DM) and characterized by structural damage and renal function decline. As the incidence of DM increases rapidly, the prevalence of DN has been increasing globally (1, 2). However, no specific treatment is available for DN to prevent the progression of end-stage renal disease (ESRD) (3). Thus, exploring new potential risk factors for the long-term prognosis of DN is important.

Type 2 DM (T2DM)-related DN is a metabolic disorder essentially that can also manifest as local inflammation of the kidney, a classic symbol of driving fibrosis and structural remodeling (4, 5). No clinical intervention was specifically targeting inflammatory mediators that could delay the progression of DN (6). Moreover, existing relevant study on the immune mechanism of DN was focused on oxidative stress, NF-κB, JAK-STAT pathways, inflammatory cytokines, or immune system-related pattern recognition receptors signaling pathways (7, 8). However, relevant clinical research about the role of inflammatory cells in DN is deficient.

Previous studies had shown that hyperglycemia could promote the activation and differentiation of T cells, and the combination of immunology and inflammatory mechanisms could play an essential role in the development and progression of T2DM (9). Some studies had shown that CD4+ T cells could promote glomerular damage, tubulointerstitial inflammatory infiltration, and loss of renal function in autoimmune glomerular disease (10, 11). Moon et al. found that the number of CD4+ T cells had increased significantly in the renal interstitial tissue of T2DM patients and was positively correlated with proteinuria. Meanwhile, significant CD4+ T cell infiltration had been observed in the kidneys of diabetic rats (12). However, the impact of CD4+ T cells on the clinical characteristics, pathomorphological changes, and long-term prognosis of DN is unclear.

In this observational cohort study, we will analyze the clinicopathological features based on the CD4 immunostaining results and then preliminarily investigate the predictive value of CD4+ T cell infiltration for renal structural damage and the prognosis of T2DM patients with DN.

## Materials and methods

### Patient selection

A total of 112 biopsy-proven DN patients with T2DM at West China Hospital of Sichuan University from September 2019 to December 2022 were reviewed. In addition, 25 patients with T2DM but pathologically diagnosed with minimal change disease were selected as the non-diabetic nephropathy (NDN) group. Patients with available pathological information were included, and patients with co-existing nondiabetic renal disease or type 1 DM were excluded. DN was defined in accordance with the criteria described by An et al. (13) and was diagnosed by at least two renal pathologists using Tervaert’s classification system (14). Indications for renal biopsy were DM with persistent albuminuria or renal dysfunction, particularly those with sudden-onset overt proteinuria or hematuria (15).

### Clinical and laboratory data collection

Baseline demographic and clinical data were collected from the electronic medical records at the time of renal biopsy, including age, sex, smoking status, body mass index (BMI), blood pressure, duration of diabetes, and use of renin–angiotensin–aldosterone system blockade, erythropoiesis-stimulating agent, and statins. Hemoglobin A1c (HbA1c), blood lipids, blood urea nitrogen, serum creatinine, uric acid, serum hemoglobin, and serum albumin were measured using routine laboratory methods with Hitachi 7600 (Hitachi, Tokyo, Japan). Simultaneously, information on 24-h proteinuria was obtained using a biochemistry autoanalyzer (CobasIntera 400 Plus, Roche, Basel, Switzerland). The estimated glomerular filtration rate (eGFR) was evaluated using the Chronic Kidney Disease Epidemiology Collaboration formula (16).

### Histologic evaluation

The biopsy specimens were processed for light microscopy, immunofluorescence, and electron microscopy. The paraffin sections for light microscopy were stained with hematoxylin and eosin, periodic acid-Schiff, and Masson’s trichrome stain for pathologic analysis. The pathological features of DN were scored according to the basis of the Renal Pathology Society (RPS) classification (14). In particular, glomerular lesion, tubular atrophy/interstitial fibrosis, interstitial inflammation, arteriolar hyalinosis, and arteriosclerosis were assessed and scored according to the RPS DN classification (14). Renal histological scoring was determined by three renal pathologists who were blinded to the clinical data and renal outcomes.

### Immunohistochemistry staining

Immunohistochemistry staining was performed using the EnVision Plus detection system (Dako, Carpinteria, CA, USA) with positive and negative controls, according to the manufacturer’s instructions, on 3-μm paraffinized sections of renal tissue. Immunostaining for CD4 (1:100, A19018; ABclonal) was performed in all 112 DN patients and 25 NDN patients. Immunostaining for Bcl6 (1:100, 66340-1-LG, Proteintech), IL-6 (1:200, GB11117, Servicebio), STAT3 (1:100, ab68153, abcam), and TGFβ1 (1:100, bs-20411R, Bioss) was performed in five patients selected randomly from the high-CD4 group, low-CD4 group, and NDN group, respectively. Semi-quantitative analysis of immunohistochemical results (%) was performed using Image J software. Five regions with relatively strong staining under high-power field (×200) were randomly collected from each case, and the average relative expression level was calculated. The DN specimens were divided into high-CD4 and low-CD4 groups by binary method based on CD4 immunostaining results. All immunohistochemistry assessments were performed by two renal pathologists, and they were blinded to the grouping.

### Analysis of mitochondrial morphology

Mitochondrial morphology was assessed by transmission electron microscopy. Up to five images of proximal tubule epithelial cells per kidney section were randomly collected for each patient at ×6,000 magnification. The mitochondrial length and width of all mitochondria within a given image were measured using Image J, and the aspect ratio (length/width) was calculated and expressed as mean aspect ratio per patient (17). In addition, the number of mitochondria within each image was evaluated and expressed as mean mitochondrial number per patient.

### Renal outcomes

All DN patients were regularly followed up for at least 12 months until December 2023. Presence of renal endpoint events, death, or loss to follow-up was the end of follow-up. The composite endpoint events of kidney was the doubling of baseline serum creatinine (D-SCr) level and/or progression to ESRD, which was defined by e-GFR <15 mL/min/1.73 m^2^ or commencing renal replacement therapy (16).

### Statistical analysis

Statistical analysis was performed using SPSS 26.0 software or GraphPad Prism version 9.5 (GraphPad Software, San Diego, CA, USA). Normally distributed continuous variables were expressed as means ± standard deviations (SDs), non-normally distributed continuous variables were expressed as median (25th–75th percentiles), and categorical variables were expressed as a number (percentage). One-way ANOVA with Tukey’s post-test was used to determine statistical significance among the high-CD4, low-CD4, and NDN groups where nonparametric data were logarithmically transformed before analysis. Morphological characteristics and mitochondrial damage were analyzed to determine the differences between the high-CD4 and low-CD4 groups using *t*-test or Mann–Whitney’s *U*-test as appropriate. Categorical variables were analyzed using chi-square test or Fisher’s exact test. Survival curves were generated using the Kaplan–Meier method, and log-rank test was performed between the high-CD4 and low-CD4 DN groups. Multivariate Cox proportional hazards models were used to determine the factors associated with the risk of reaching composite endpoint of kidney. The results were expressed as hazard-rate (HRs) with 95% CI. A value of *p* <0.05 was considered as a significant difference.

## Results

### Demographic and clinical characteristics according to the infiltration of CD4+ T cells at the time of biopsy

The cohort of 112 patients with DN was stratified according to the immunostaining results of CD4 in biopsied specimens as either high-CD4 group (*n* = 56, median semi-quantitative CD4 expression: 24.4%) or low-CD4 group (*n* = 56, median semi-quantitative CD4 expression: 7.9%). The infiltration of CD4+ T cells in the renal tissue of NDN patients (median semi-quantitative CD4 expression: 4.8%) was significantly lower than that of DN patients (*p* < 0.001). The demographic and clinical characteristics of the patients are displayed in **Table 1**.

**Table 1:** Clinical characteristics of DN patients grouped accorrding to the results of CD4 immunohistochemistry staining (NDN patients as control group).

Characteristics	DN patients (All, n=112)	HIGH-CD4 (DN patients, n=56)	LOW-CD4 (DN patients, n=56)	NDN patients (n=25)	*p*
CD4 expression, median (IQR), (%)	13.1 (7.9-24.4)	24.4 (18.1-32.5)	7.9 (5.3-10.8)	4.8 (2.4-9.4)	<0.001
Age, mean (SD), y	49.2 (9.4)	48.8 (9.5)	49.6 (9.3)	51.4 (9.3)	0.509
Sex, male, n (%)	86 (76.8%)	41 (73.2%)	45 (80.4%)	15 (60%)	0.156
Smoking, Current/Ex/Never, (n)	25/14/73	10/9/37	15/5/36	3/1/21	0.208
BMI, mean (SD), kg/m2	24.7 (3.5)	24.3 (3.6)	25.1 (3.4)	25.5 (4.2)	0.294
SBP, mean (SD), mmHg	143.8 (22.8)	144.6 (23.3)	143 (22.6)	128 (19.3)	0.016
DBP, mean (SD), mmHg	88.6 (14.3)	88.3 (14.1)	88.8 (14.7)	86.1 (12.6)	0.756
Duration of diabetes, median (IQR), months	120 (60-144)	120 (60-144)	96 (48-144)	88.5 (83.5-93)	<0.001
HbA1c, median (IQR), %	7.7 (6.45-9.15)	7.5 (6.45-8.45)	7.95 (5.7-11.2)	6.1 (5.75-7.1)	0.015
Serum Hb, mean (SD), g/dL	115.1 (24.1)	109.7 (24.4)	120.6 (22.8)	139.6 (15.9)	<0.001
Serum albumin, mean (SD), g/L	31.9 (6.99)	32.3 (6.5)	31.4 (7.5)	40.5 (9.4)	<0.001
eGFR, mean (SD), mL/min/1.73 m^2^	51.4 (26.6)	43.3 (25.4)	59.6 (25.5)	88.8 (24.9)	<0.001
Serum creatinine, mean (SD), umol/L	171.7 (108.3)	203.9 (129.6)	138.8 (67.9)	86.2 (49.2)	<0.001
24-h proteinuria, mean (SD), g/d	7.2 (5.96)	7.7 (6.2)	6.7 (5.7)	2.5 (3.3)	0.003
Urinary Albumin to Creatinine Ratio, mean (SD), mg/mmol	3133.5 (2168.7)	3383.3 (2209.2)	2839.1 (2121.6)	1213.2 (1757.1)	0.007
Uric acid, mean (SD), mg/dL	384.3 (90.1)	383.2 (91.0)	385.3 (90.1)	348.9 (105.7)	0.245
Triglyceride, median (IQR), mg/dL	1.7 (1.2-2.6)	1.7 (1.2-2.4)	1.7 (1.2-2.8)	1.8 (1.1-3.8)	0.031
Cholesterol, mean (SD), mg/dL	5.4 (2.1)	5.3 (2.3)	5.6 (2.0)	5.2 (2.3)	0.674
HDL-C, mean (SD), mg/dL	1.2 (0.5)	1.1 (0.48)	1.2 (0.48)	1.2 (0.48)	0.640
LDL-C, mean (SD), mg/dL	3.4 (1.8)	3.3 (1.9)	3.5 (1.7)	3.0 (1.9)	0.530
RAAS inhibitors, n (%)	64 (57.1%)	31 (55.4%)	33 (58.9%)	14 (56%)	0.925
Statins, n (%)	55 (49.1%)	29 (51.8%)	26 (46.4%)	7 (28%)	0.135
ESA, n (%)	11 (9.8%)	8 (14.3%)	3 (5.4%)	0 (0)	0.058

Data are presented as the mean (standard) for continuous variables with symmetric distribution, median (25th–75th percentiles) for continuous variables with asymmetric distribution, or percentages for categorical variables.

†Defined by RPS Diabetic Nephropathy Classification.

BMI, body mass index; DBP, diastolic blood pressure; eGFR, estimated glomerular filtration rate; ESA, erythropoiesis- stimulating agent; Hb, Hemoglobin; HbA1c, hemoglobin A1c; HDL-C, high density lipoprotein-cholesterol; IQR, interquartile range; LDL-C, low density lipoprotein-cholesterol; RAAS, renin-angiotensin- aldosterone system; RPS, Renal Pathology Society; SBP, systolic blood pressure; SD, standard deviation.

The baseline characteristics of the study population was presented with no significant differences among the high-CD4 group, low-CD4 group, and NDN patients in terms of sex, age, smoking status, BMI, diastolic blood pressure, serum uric acid, serum cholesterol, and treatment. Compared with the NDN patients and the patients in the low-CD4 group, the patients in the high-CD4 group suffered from a lower eGFR level (high-CD4: 43.3 mL/min/1.73 m^2^ vs. low-CD4: 59.6 mL/min/1.73 m^2^ vs. NDN: 88.8 mL/min/1.73 m^2^, *p* < 0.001), a higher degree of proteinuria (high-CD4: 7.7 g/day vs. low-CD4: 6.7 g/day vs. NDN: 2.5 g/day, *p* = 0.003), a higher serum creatinine level (high-CD4: 203.9 umol/L vs. low-CD4: 138.8 umol/L vs. NDN: 86.2 umol/L, *p* < 0.001), and a higher urinary-albumin-to-creatinine ratio (high-CD4: 3,383.3 mg/mmol vs. low-CD4: 2,839.1 mg/mmol vs. NDN: 1,213.2 mg/mmol, *p* = 0.007). Significant differences among these three groups were also found for systolic blood pressure, duration of diabetes, HbA1c, serum hemoglobin, serum albumin, and serum triglyceride.

### DN patients in the high-CD4 group presented with severe pathological lesions and suffered from more severe mitochondrial damage of renal tubular epithelial cells

**Figure 1:**
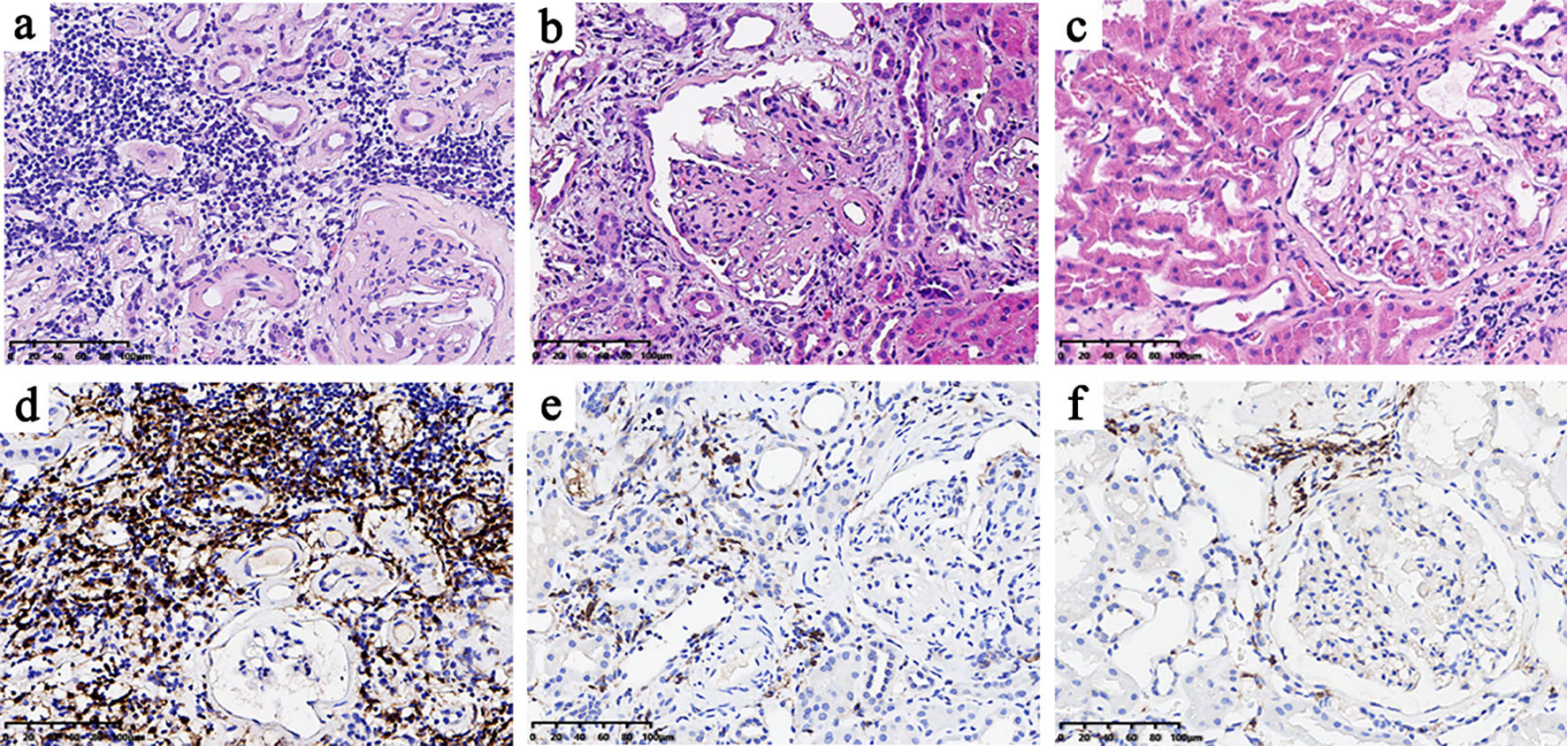
Histological features of the high-CD4 and low-CD4 groups **(A–C)** and CD4 immunohistochemical results **(D–F)**. The histological features of the high-CD4 group **(A, D)** showed heavier interstitial inflammatory cell infiltration, more severe renal tubular atrophy, and interstitial fibrosis than those in the low-CD4 group **(B, E)** and non-diabetic nephropathy group **(C, F)**.

The representative morphological characteristics of light microscopy are shown in **Figure 1**. DN patients harbored more severe interstitial inflammation infiltration and tubular atrophy/interstitial fibrosis than NDN patients. Consistent with the lower eGFR and higher proteinuria levels, DN patients in the high-CD4 group presented with a higher percentage of severe glomerular injury (RPS grades III and IV, 75% vs. 53.6%, *p* = 0.007), severe tubular atrophy/interstitial fibrosis (grades 2 and 3, 78.6% vs. 42.9%, *p* = 0.001; mean area %, 39.6% vs. 23.3%, *p* < 0.001), severe interstitial inflammation infiltration (grade 2, 87.5% vs. 57.1%, *p* < 0.001), more arteriosclerosis (grade 2, 48.2% vs. 26.8%, *p =* 0.022), and severe arteriolar hyalinosis (grade 2, 78.5% vs. 37.5%, *p* < 0.001) than DN patients in the low-CD4 group (**Table 2**).

**Table 2:** Renal pathological characteristics of DN patients grouped accorrding to the results of CD4 immunohistochemistry staining.

Characteristics	DN patients (All, n=112)	HIGH-CD4 (DN patients, n=56)	LOW-CD4 (DN patients, n=56)	*p*
RPS glomerular lesion† , n (%)				0.007
I	0 (0)	0 (0)	0 (0)	
IIa	18 (16.1)	3 (5.4)	15 (26.8)	
IIb	22 (19.6)	11 (19.6)	11 (19.6)	
III	62 (55.4)	34 (60.7)	28 (50)	
IV	10 (8.9)	8 (14.3)	2 (3.6)	
Tubular atrophy/Interstitial fbrosis, mean (SD), (%)	31.5 (21.2)	39.6 (18.5)	23.3 (20.8)	<0.001
Tubular atrophy/Interstitial fbrosis† n (%)				0.001
Score 0	3 (2.7)	0 (0)	3 (5.3)	
Score 1	41 (36.6)	12 (21.4)	29 (51.8)	
Score 2	37 (33)	22 (39.3)	15 (26.8)	
Score 3	31 (27.7)	22 (39.3)	9 (16.1)	
Interstitial infammation† n (%)				<0.001
Score 0	0 (0)	0 (0)	0 (0)	
Score 1	31 (27.7)	7 (12.5)	24 (42.9)	
Score 2	81 (72.3)	49 (87.5)	32 (57.1)	
Arteriosclerosis†, n (%)				0.022
Score 0	3 (2.7)	0 (0)	3 (5.4)	
Score 1	67 (59.8)	29 (51.8)	38 (67.8)	
Score 2	42 (37.5)	27 (48.2)	15 (26.8)	
Arteriolar hyalinosis†, n (%)				<0.001
Score 0	11 (9.8)	3 (5.4)	8 (14.3)	
Score 1	36 (32.2)	9 (16.1)	27 (48.2)	
Score 2	65 (58)	44 (78.5)	21 (37.5)	

Data are presented as the mean (standard) for continuous variables with symmetric distribution, or percentages for categorical variables. RPS, Renal Pathology Society; SD, standard deviation.

†Defned by RPS Diabetic Nephropathy Classifcation.

Univariate logistic regression analyses showed that RPS glomerular lesion (OR 2.60, 95% CI 1.17–5.79, *p* = 0.019), tubular atrophy/interstitial fibrosis (OR 4.89, 95% CI 2.13–11.20, *p* < 0.001), interstitial inflammation (OR 5.25, 95% CI 2.03–13.61, *p* = 0.001), arteriosclerosis (OR 2.55, 95% CI 1.16–5.61, *p* = 0.021), and arteriolar hyalinosis (OR 6.11, 95% CI 2.65–14.11, *p* < 0.001) were associated with high-CD4 staining. However, the multivariate logistic regression analyses showed that only tubular atrophy/interstitial fibrosis (OR 6.20, 95% CI 1.92–19.98, *p* = 0.002), interstitial inflammation (OR 12.38, 95% CI 2.83–54.13, *p* = 0.001), and arteriolar hyalinosis (OR 5.51, 95% CI 1.99–15.29, *p* = 0.001) were significantly associated with high CD4 staining. In addition, both univariate and multivariate logistic regression analyses showed that tubular atrophy/interstitial fibrosis, interstitial inflammation, arteriosclerosis, and arteriolar hyalinosis were significantly associated with the semi-quantitative percentage of CD4 immunostaining (**Table 3**).

**Table 3:** Univariable and multivariable logistic proportional hazard models analysis of the effect of CD4+T cell infiltration on renal pathological changes.

Pathological characteristics	Percentage of CD4 immunostaining (%)						High CD4 immunostaining (binary analysis)					
	Univariate			Multivariate			Univariate			Multivariate		
	Odds ratio	(95% CI)	p	Odds ratio	(95% CI)	p	Odds ratio	(95% CI)	p	Odds ratio	(95% CI)	p
RPS glomerular lesion (graded III and IV)	1.02	0.99-1.06	0.136	1.02	0.98-1.06	0.300	2.60	1.17-5.79	0.019	2.33	0.88-6.15	0.087
Tubular atrophy/Interstitial fbrosis (score>1)	1.12	1.06-1.19	<0.001	1.13	1.06-1.21	<0.001	4.89	2.13-11.20	<0.001	6.20	1.92-19.98	0.002
Interstitial inflamation (score>1)	1.13	1.05-1.20	<0.001	1.13	1.05-1.22	0.001	5.25	2.03-13.61	0.001	12.38	2.83-54.13	0.001
Arteriosclerosis (score>1)	1.03	1.00-1.06	0.030	1.04	1.00-1.07	0.047	2.55	1.16-5.61	0.021	2.04	0.76-5.48	0.158
Arteriolar hyalinosis (score>1)	1.13	1.07-1.19	<0.001	1.13	1.06-1.21	<0.001	6.11	2.65-14.11	<0.001	5.51	1.99-15.29	0.001

CI, confdence interval; RPS, Renal Pathology Society.

These variables were adjusted in multivariate logistic regression analyses: Age, gender, and clinical variables that are statistically different between the two groups and that might affect renal pathological changes include SBP, duration of diabetes, HbA1c, serum Hb, serum albumin and triglyceride.

**Figure 2:**
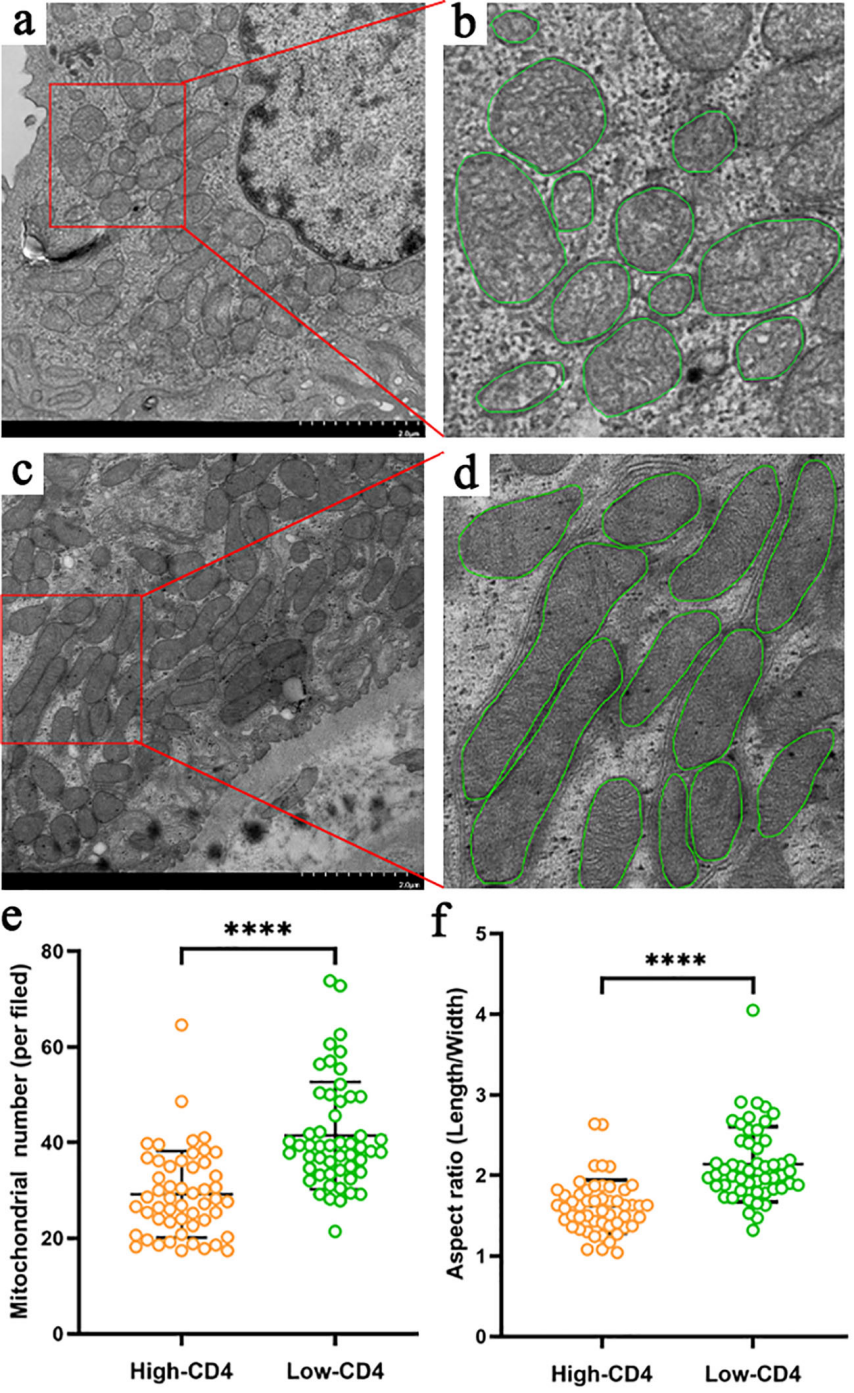
Characteristics of mitochondria in proximal tubular epithelial cells of diabetic nephropathy patients grouped by CD4 immunostaining results **(A–F)**. Compared with the patients in the low-CD4 group **(C, D)**, patients in the high-CD4 group **(A, B)** harbored more severe mitochondrial damage manifested by more obvious rupture of the mitochondrial cristae, fewer mitochondrial number **(E)**, and more obvious mitochondrial swelling (smaller aspect ratio) **(F)**. ****p < 0.0001.

We finally analyzed the mitochondrial morphology of renal tubular epithelial cells under an electron microscope in 104 DN patients with appropriate specimens (52 in the high-CD4 group and 52 in the low-CD4 group) (**Figures 2A–F**). Notably, obvious mitochondrial swelling and mitochondrial cristae rupture had been observed in DN patients. The mitochondrial count was fewer (29 ± 9 vs. 41 ± 11, *p* < 0.001), the aspect ratio was smaller (1.6 ± 0.3 vs. 2.1 ± 0.5, *p* < 0.001), and the mitochondrial cristae rupture was more severe in the high-CD4 group (**Figures 2A, B**) than those in the low-CD4 group (**Figures 2C, D**).

### Increased expression of molecules associated with renal tubular atrophy/interstitial fibrosis in the high-CD4 group of DN patients

**Figure 3:**
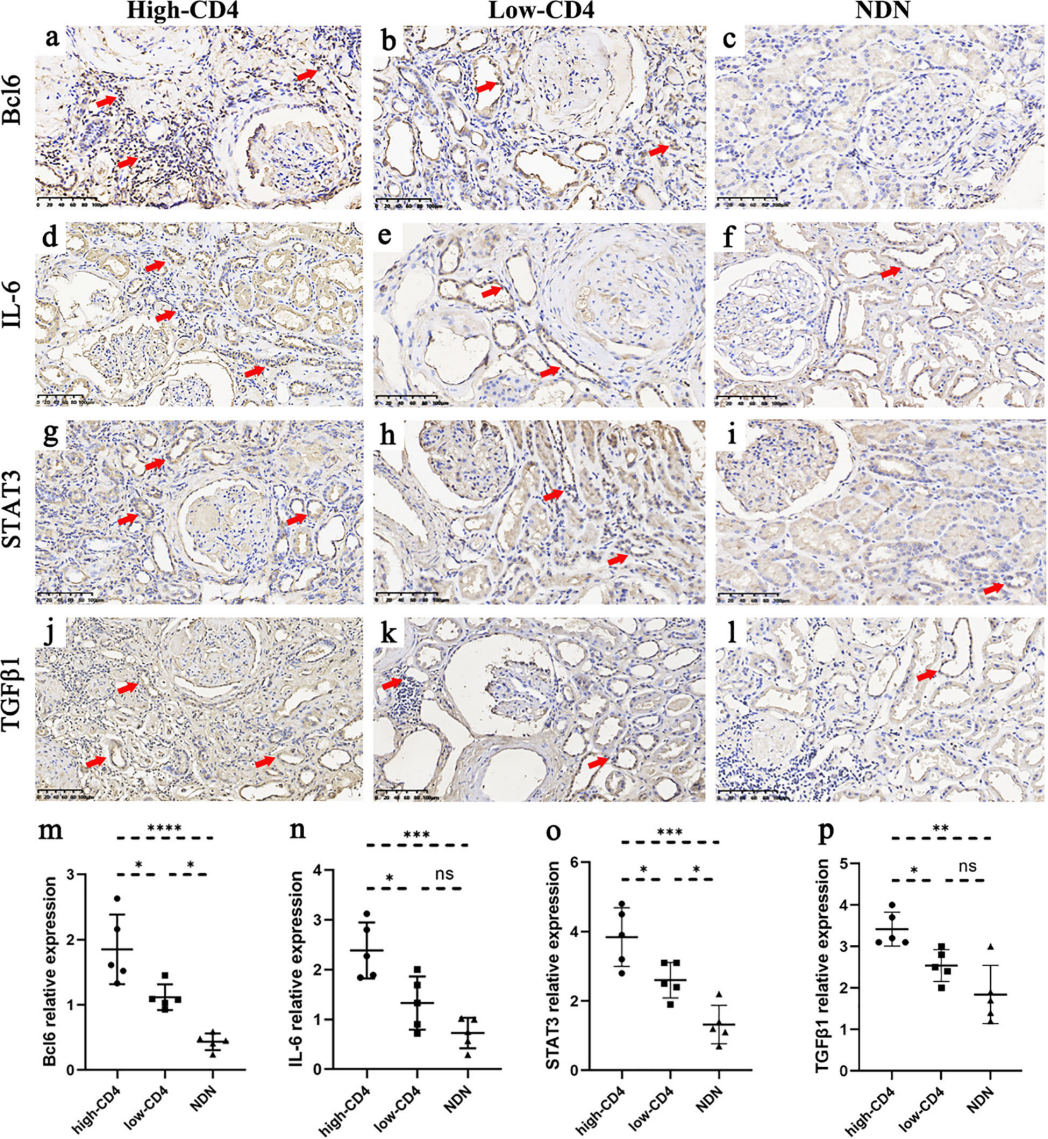
Immunostaining results of some molecules among different groups. **(A–L)** Immunohistochemical results of Bcl6 **(A–C)**, IL-6 **(D–F)**, STAT3 **(G–I)**, and TGFβ1 **(J–L)** in the high-CD4, low-CD4, and non-diabetic nephropathy (NDN) groups (scale bar = 100 μm; red arrows indicate partial positive expression). **(M–P)** The expression of Bcl6, IL-6, STAT3, and TGFβ1 in renal tissue was more obvious in the high-CD4 group than in the low-CD4 and NDN groups (data was shown as mean ± SD, five cases in each group; ns, not significant; *p < 0.05, **p < 0.01, ***p < 0.001).

To understand renal tubular atrophy, interstitial fibrosis, and CD4+ T cell infiltration among these patients, we had detected the expression of the related molecules such as Bcl6, IL-6, STAT3, and TGFβ1 in renal biopsies. The immunohistochemical results showed that the positive expression of the molecules was mainly in renal tubular epithelial cells and renal interstitial cells (**Figures 3A–L**). We had observed that DN patients in the high-CD4 group significantly presented with a relatively higher expression level of Bcl6, IL-6, STAT3, and TGFβ1 than DN patients in the low-CD4 group and NDN patients (**Figures 3M–P**, *p* < 0.05).

### Prognostic value of CD4+ T cell infiltration for renal outcome in patients with DN

**Figure 4:**
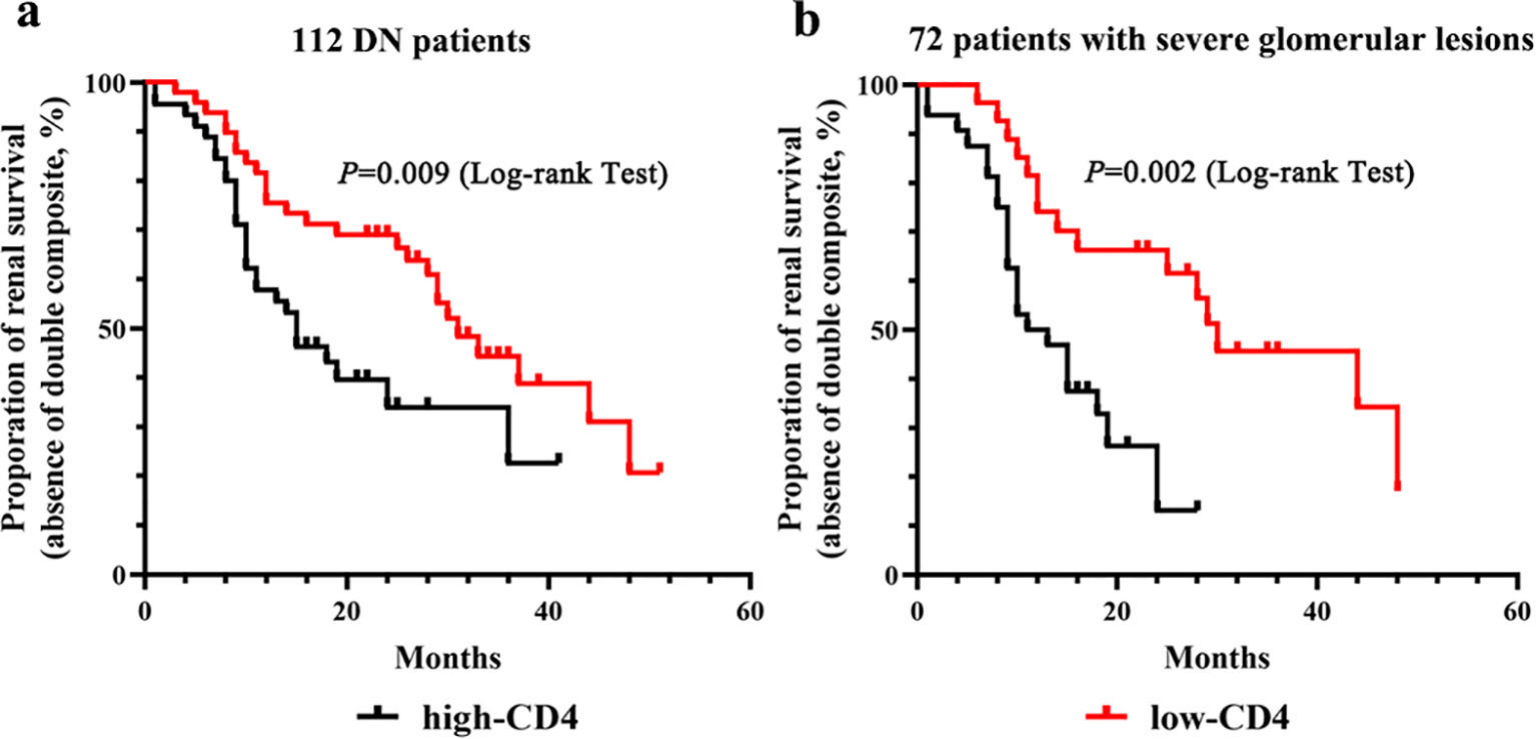
Kaplan–Meier survival curves for renal outcome according to the CD4 immunostaining results. **(A)** Kaplan–Meier survival curves for renal outcome stratified by the severity of CD4+ T cell infiltration in the total of 112 diabetic nephropathy patients. **(B)** Kaplan–Meier survival curves for renal outcome stratified by the severity of CD4+ T cell infiltration in 72 patients with severe glomerular lesions.

All 112 DN patients were followed up for 12 to 51 months until a renal endpoint event occurred, including 13 patients lost to follow-up (11.6%). The composite endpoint occurred in 59.6% (59/99) patients. Survival curves were performed according to the Kaplan–Meier method. In the high-CD4 group, 65.3% (32/49) patients experienced endpoint events, and the median survival time was 15 months. In the low-CD4 group, endpoint events were observed in 54% (27/50) patients, with a median survival time of 31 months (*p* = 0.009). The proportion of renal survival was 20.7% at 51 months of follow-up in the low-CD4 group and 22.6% at 41 months of follow-up in the high-CD4 group (**Figure 4A**). Among DN patients with severe glomerular lesions, the median survival time was 12 months for patients in the high-CD4 group and 30 months for patients in the low-CD4 group (*p* = 0.002). The proportion of renal survival was 17.1% at 48 months of follow-up in the low-CD4 group and 13.1% at 28 months of follow-up in the high-CD4 group (**Figure 4B**).

We then investigated whether CD4+ T cell infiltration could independently predict poor renal outcome (**Table 4**). In the univariate Cox regression model, renal outcome for composite endpoint was significantly associated with CD4 immunostaining. After adjusting for baseline parameters plus parameters with statistical differences among groups, the higher-CD4 immunostaining remained as an independent predictor for renal endpoint events (**Table 4**, model 1; adjusted HR 2.61, 95% CI 1.36–5.03, *p* = 0.004). When the histopathologic features were included in the multivariate model, the higher-CD4 immunostaining was still significantly associated with adverse renal outcomes (**Table 4**, model 2; adjusted HR 3.17, 95% CI 1.28–7.90, *p* = 0.013). Otherwise, the percentage of positive CD4 staining remained as the risk factor for poor renal prognosis after adjusting for clinical, pathological, and renal function parameters (**Table 4**, model 3; adjusted HR 1.80, 95% CI 1.21–2.68, *p* = 0.003).

**Table 4:** Univariable and multivariable Cox analysis of the effect of CD4+T cell infiltration on renal prognosis.

Variables	CD4 immunostaining (binary analysis)		Percentage of CD4 immunostaining (%)
	Low-CD4	High-CD4	
Univariate Models HR (95% CI)	1 (reference)	2.35 (1.34-4.13)	1.03 (1.00-1.04)
*p value*	0.003		0.006
Multivariable a model 1^a^ HR (95% CI)	1 (reference)	2.61 (1.36-5.03)	1.03 (1.01-1.06)
*p value*	0.004		0.002
Multivariable b model 2^b^ HR (95% CI)	1 (reference)	3.17 (1.28-7.90)	1.03 (1.00-1.06)
*p value*	0.013		0.023
Multivariable b model 3^c^ HR (95% CI)	1 (reference)	4.73 (0.21-104.62)	1.80 (1.21-2.68)
*p value*	0.325		0.003

^a^ Adjusted for baseline parameters age and sex, plus parameters with statistical differences including systolic blood pressure, duration of diabetes, hemoglobin A1c, hemoglobin, serum albumin, and triglyceride.

^b^ Adjusted for the parameters in multivariable model a plus pathological parameters (Renal Pathology Society glomerular classifcations, interstitial fbrosis and tubular atrophy, interstitial infammation, arteriosclerosis and arteriolar hyalinosis).

^b^ Adjusted for the parameters in multivariable model b plus Clinical renal function parameters (eGFR, Serum creatinine, 24-h proteinuria, Urinary Albumin to Creatinine Ratio).

## Discussion

In this study, we found that patients with heavier CD4+ T cell infiltration suffered from higher proteinuria and lower eGFR levels, more severe glomerular lesions, heavier renal interstitial inflammatory infiltration, more severe tubular atrophy/interstitial fibrosis, more severe arterial hyalinosis, and more severe mitochondrial injury. According to the follow-up data, a higher density of CD4+ T cell infiltration was associated with deterioration of renal function in T2DM patients with DN.

This study had shown that the degree of CD4+ T cell infiltration in the renal tissue of DN patients was significantly higher than that in NDN control patients. A previous study had also reported that the penetration level of CD4 + effective memory T cells in the kidneys of DN was significantly increased compared to healthy controls by using xCell to generate cell type enrichment scores from gene expression data (18). This study had found that the heavy infiltration of CD4+ T cells was associated with the decrease of eGFR and the increase of serum creatinine and proteinuria levels. The close relationship between CD4+ T cell infiltration in renal interstitium and the presence of proteinuria had been likewise observed previously (12). All of the above indicated that CD4+ T cells might play a non-negligible role in the occurrence and progression of renal injury of T2DM patients.

In immune-mediated glomerular diseases, CD4+ T cells would be accumulated within the tubulointerstitial compartment in close contact to proximal and distal tubular epithelial cells and drive renal inflammation and tissue damage (19). Another study had implicated that CD4+ T cells were involved in interstitial fibrosis to induce kidney injury (20). Otherwise, the results in this study had shown that CD4+ T cell infiltration was proportional to the degree of renal tubular atrophy and interstitial fibrosis and was related to the prognosis of DN. The associated mechanism of CD4+ T cells highly related to renal injury of DN might be the activation of the JAK/STAT pathway after upregulation of CD4+ T cells or the activation of cytokines such as TGFβ, IL6, and STAT3 (21). Our immunohistochemical analysis results also supported this conclusion. IL-6 and TGFβ1 could synergistically promote the differentiation of T cells, thereby increasing Bcl6 levels, and at the same time activate the STAT3 pathway (22). However, the sample size was limited, and the analysis was based on semiquantitative results. More molecular research is desired to confirm this mechanism hypothesis. Nevertheless, a previous study had shown that both the classical and the trans-signaling pathways of IL-6 were shown to participate in the pathogenesis and progression of DN (21). IL-6 trans-signaling and phosphorylation of STAT3 were associated with tubular atrophy and interstitial fibrosis (23). STAT3 was mechanistically involved in the communication between epithelial cells and fibroblasts (24), and IL-6-STAT3 signaling could enhance the expression of TGF-β1 to promote fibrosis (25). In addition, an experiment data in mouse had demonstrated that proximal tubular cells could activate CD4+ T cells through antigen presentation, leading to T cell spread and inflammatory cytokine secretion (26). The binding of CD4+ T cells to MHC class II antigens would serve as an important role in immune effects and promoting renal fibrosis (26). Of course, the effect of CD4+ T cells on renal structural damage of DN and associated mechanism still requires further investigation.

CD4+ T cells included a cluster of highly plastic Th cells, primarily grouped into Th1, Th2, Th3, Th9, Th17, Th22, T follicular helper, and Tregs (27). CD4+ T cell subsets could obtain regulatory functions under chronic high glucose stimulation, opening up a new perspective for exploring the immune regulation mechanism of DM (27, 28). Compared with healthy controls, the number of CD4 + CXCR5 +T cells in the peripheral blood of DN patients was increased, which was proportional to the 24-h urine protein level, and the number of CD4 + CXCR5 +T cells was decreased after the patients’ routine treatment (29). Besides that, Kim et al. found that CD4+ T cells in the kidneys of diabetic mice increased significantly since the appearance of proteinuria (30). Interestingly, mycophenolate mofetil only suppressed IL-17+CD4+ T cells in the kidney of early DN and could improve albuminuria and interstitial fibrosis independent of glycemic control (30). Further research is desired to explore the specific targets to balance CD4+ T cells in the immunotherapy of DN.

Notably, it had been reported that the injury to proximal tubular cells occurred before glomerular podocytes in a diabetic rat model, which meant that renal tubular damage might be the initiating factor of DN (31). Proximal tubular mitochondria were the crucial organelles regulating oxidative stress and hypoxic damage in diabetic conditions (32), and mitochondrial dysfunction induced by high glucose was involved in the development of DN (33, 34). This study supported the phenomenon that CD4+ T cell infiltration in kidney tissue was associated with mitochondrial injury in renal tubular epithelial cells of DN. Of course, further research is desired to clarify the mechanism about CD4+ T cells and mitochondrial damage.

Some previous studies indicated that inflammation was one of the pathogenesis of DN. The interaction between T cells, macrophages, dendritic cells, and renal tubular cells might be one of the mechanisms for inflammation mediating the progression of DN (5, 35). Lim et al. showed that, among STZ-induced mice, only Rag1 (+/+) mice with mature T lymphocytes developed globular immunoglobulin deposition (36). In addition, the extent of albuminuria was regulated by the number of T cells in the kidneys of DN, and Abatacept had been observed to improve DN by blocking the activation of systemic T cells (37). Our results also supported that CD4+ T cells were associated with renal pathological injury in DN. However, this phenomenon needs to be viewed dialectically. Abais et al. showed that STZ-induced mice lacking T cells had higher serum tumor necrosis factor, albuminuria, and more hypertrophy of the kidneys than wild-type mice (38). Therefore, more experimental evidence is needed to demonstrate how CD4+ T cells affect DN.

This study had certain limitations. First, the sample size was relatively limited; more research on a larger population is needed to harbor generalizability of the findings. Second, the NDN patients were included as the control in clinical characteristics and molecular analysis in order to show the influence of CD4+ T cells on the occurrence of DN, but only the high-CD4 and low-CD4 groups of DN patients in pathological and prognostic analysis were compared due to the differences in assessment standards for different renal diseases. It might be beneficial to include a broader range of control groups for comparison. Third, the research method in this study was mainly based on semi-quantitative analysis of immunohistochemical results, and there were inevitable misclassification biases. Future studies should aim to refine these methods. Fourth, we had analyzed the mitochondrial morphology in renal tubular epithelial cells in this study. Nonetheless, the podocytes, mesangial cells, and interstitial fibroblasts should also be observed in future research.

In summary, this study highlighted the clinical significance of CD4+ T cell infiltration in the renal interstitium for the first time and indicated that the high density of CD4+ T cell distribution was associated with severe glomerular lesions, tubular atrophy, and interstitial fibrosis. Notably, CD4+ T cell infiltration was significantly associated with poor renal outcome in DN patients. Moreover, mitochondrial dysfunction in renal tubular epithelial cells might be an intermediate mechanism in which CD4+ T cells participated in kidney injury and renal function decline of DN patients. These findings suggested that CD4+ T cells are potentially served as a therapeutic target for DN patients. Nevertheless, further cellular and animal experiments are still needed to verify the clinical findings.

## Data Availability

The original contributions presented in the study are included in the article/supplementary material. Further inquiries can be directed to the corresponding authors.

## References

[1] O'Shaughnessy MM Hogan SL Poulton CJ Falk RJ Singh HK Nickeleit V. Temporal and demographic trends in glomerular disease epidemiology in the southeastern United States, 1986-2015. Clin J Am Soc Nephrol. (2017) 12:614–23. doi: 10.2215/CJN.10871016 PMC5383393 28325866

[2] Han Q Xu H Li L Lei S Yang M. Demographic distribution analysis of different glomerular diseases in Southwest China from 2008 to 2022. Int Urol Nephrol. (2024) 56:2011–20. doi: 10.1007/s11255-023-03902-9 38172368

[3] Selby NM Taal MW. An updated overview of diabetic nephropathy: Diagnosis, prognosis, treatment goals and latest guidelines. Diabetes Obes Metab. (2020) 22 Suppl 1:3–15. doi: 10.1111/dom.14007 32267079

[4] Umanath K Lewis JB. Update on diabetic nephropathy: core curriculum 2018. Am J Kidney Dis. (2018) 71:884–95. doi: 10.1053/j.ajkd.2017.10.026 29398179

[5] Zheng Z Zheng F. Immune cells and inflammation in diabetic nephropathy. J Diabetes Res. (2016) 2016:1841690. doi: 10.1155/2016/1841690 26824038 PMC4707326

[6] Rayego-Mateos S Rodrigues-Diez RR Fernandez-Fernandez B Mora-Fernández C Marchant V Donate-Correa J. Targeting inflammation to treat diabetic kiDKDey disease: the road to 2030. KiDKDey Int. (2023) 103:282–96. doi: 10.1016/j.kint.2022.10.030 36470394

[7] Wada J Makino H. Innate immunity in diabetes and diabetic nephropathy. Nat Rev Nephrol. (2016) 12:13–26. doi: 10.1038/nrneph.2015.175 26568190

[8] García-García PM Getino-Melián MA Domínguez-Pimentel V Navarro-González JF. Inflammation in diabetic kiDKDey disease. World J Diabetes. (2014) 5:431–43. doi: 10.4239/wjd.v5.i4.431 PMC4127580 25126391

[9] Duran-Salgado MB Rubio-Guerra AF. Diabetic nephropathy and inflammation. World J Diabetes. (2014) 5:393–8. doi: 10.4239/wjd.v5.i3.393 PMC4058744 24936261

[10] Hopfer H Holzer J Hünemörder S Paust HJ Sachs M Meyer-Schwesinger C. Characterization of the renal CD4+ T-cell response in experimental autoimmune glomerulonephritis. Kidney Int. (2012) 82:60–71. doi: 10.1038/ki.2012.73 22437418

[11] Paust HJ Song N De Feo D Asada N Tuzlak S Zhao Y. CD4+ T cells produce GM-CSF and drive immune-mediated glomerular disease by licensing monocyte-derived cells to produce MMP12. Sci Transl Med. (2023) 15:eadd6137. doi: 10.1126/scitranslmed.add6137 36921033

[12] Moon JY Jeong KH Lee TW Ihm CG Lim SJ Lee SH. Aberrant recruitment and activation of T cells in diabetic nephropathy. Am J Nephrol. (2012) 35:164–74. doi: 10.1159/000334928 22286547

[13] An Y Xu F Le W Ge Y Zhou M Chen H. Renal histologic changes and the outcome in patients with diabetic nephropathy. Nephrol Dial Transpl. (2015) 30:257–66. doi: 10.1093/ndt/gfu250 25063425

[14] Tervaert TW Mooyaart AL Amann K Cohen AH Cook HT Drachenberg CB. Renal pathology S. Pathologic classification of diabetic nephropathy. J Am Soc Nephrol. (2010) 21:556–63. doi: 10.1681/ASN.2010010010 20167701

[15] Furuichi K Yuzawa Y Shimizu M Hara A Toyama T Kitamura H. Nationwide multicentre kidney biopsy study of Japanese patients with type 2 diabetes. Nephrol Dial Transpl. (2018) 33:138–48. doi: 10.1093/ndt/gfw417 28340221

[16] Han Q Wang S Zhang J Zhang R Guo R Wang Y. The association between cigarette smoking and diabetic nephropathy in Chinese male patients. Acta Diabetol. (2018) 55:1131–41. doi: 10.1007/s00592-018-1197-9 30066043

[17] Coughlan MT Higgins GC Nguyen TV Penfold SA Thallas-Bonke V Tan SM. Deficiency in apoptosis-inducing factor recapitulates chronic kidney disease via aberrant mitochondrial homeostasis. Diabetes. (2016) 65:1085–98. doi: 10.2337/db15-0864 26822084

[18] Li T Wang Y Zhu C Yang Y Long C Chen Q. Identification of Ribonuclease 6 as an immunoinflammatory key gene associated with the glomerular injury in diabetic nephropathy. Sci Rep. (2022) 12:19709. doi: 10.1038/s41598-022-24289-0 36385487 PMC9668917

[19] Breda PC Wiech T Meyer-Schwesinger C Grahammer F Huber T Panzer U. Renal proximal tubular epithelial cells exert immunomodulatory function by driving inflammatory CD4+ T cell responses. Am J Physiol Renal Physiol. (2019) 317:F77–89. doi: 10.1152/ajprenal.00427.2018 31017008

[20] Meng XM Nikolic-Paterson DJ Lan HY. Inflammatory processes in renal fibrosis. Nat Rev Nephrol. (2014) 10:493–503. doi: 10.1038/nrneph.2014.114 24981817

[21] Feigerlová E Battaglia-Hsu SF. IL-6 signaling in diabetic nephropathy: From pathophysiology to therapeutic perspectives. Cytokine Growth Factor Rev. (2017) 37:57–65. doi: 10.1016/j.cytogfr.2017.03.003 28363692

[22] Crotty S. T follicular helper cell differentiation, function, and roles in disease. Immunity. (2014) 41:529–42. doi: 10.1016/j.immuni.2014.10.004 PMC4223692 25367570

[23] Chen W Yuan H Cao W Wang T Chen W Yu H. Blocking interleukin-6 trans-signaling protects against renal fibrosis by suppressing STAT3 activation. Theranostics. (2019) 9:3980–91. doi: 10.7150/thno.32352 PMC6592178 31281526

[24] Bienaime F Muorah M Yammine L Burtin M Nguyen C Baron W. Stat3 controls tubulointerstitial communication during CKD. J Am Soc Nephrol. (2016) 27:3690–705. doi: 10.1681/ASN.2015091014 PMC5118479 27153926

[25] O'Reilly S Ciechomska M Cant R van Laar JM. Interleukin-6 (IL-6) trans signaling drives a STAT3-dependent pathway that leads to hyperactive transforming growth factor-beta (TGF-beta) signaling promoting SMAD3 activation and fibrosis via Gremlin protein. J Biol Chem. (2014) 289:9952–60. doi: 10.1074/jbc.M113.545822 PMC3975039 24550394

[26] Zhou Y Luo Z Liao C Cao R Hussain Z Wang J. MHC class II in renal tubules plays an essential role in renal fibrosis. Cell Mol Immunol. (2021) 18:2530–40. doi: 10.1038/s41423-021-00763-z PMC8545940 34556823

[27] Liu Y Lv Y Zhang T Huang T Lang Y Sheng Q. T cells and their products in diabetic kidney disease. Front Immunol. (2023) 14:1084448. doi: 10.3389/fimmu.2023.1084448 36776877 PMC9909022

[28] Soukou S Huber S Krebs CF. T cell plasticity in renal autoimmune disease. Cell Tissue Res. (2021) 385:323–33. doi: 10.1007/s00441-021-03466-z PMC8088832 33937944

[29] Zhang N Tai J Qu Z Zhang Z Zhao S He J. Increased CD4+CXCR5+T follicular helper cells in diabetic nephropathy. Autoimmunity. (2016) 49:405–13. doi: 10.1080/08916934.2016.1196677 27477820

[30] Kim SM Lee SH Lee A Kim DJ Kim YG Kim SY. Targeting T helper 17 by mycophenolate mofetil attenuates diabetic nephropathy progression. Transl Res. (2015) 166:375–83. doi: 10.1016/j.trsl.2015.04.013 26001596

[31] Coughlan MT Nguyen TV Penfold SA Higgins GC Thallas-Bonke V Tan SM. Mapping time-course mitochondrial adaptations in the kiDKDey in experimental diabetes. Clin Sci (Lond). (2016) 130:711–20. doi: 10.1042/CS20150838 26831938

[32] Lee WC Chau YY Ng HY Chen CH Wang PW Liou CW. Empagliflozin protects HK-2 cells from high glucose-mediated injuries via a mitochondrial mechanism. Cells. (2019) 8:1085. doi: 10.3390/cells8091085 31540085 PMC6770192

[33] Czajka A Ajaz S Gnudi L Parsade CK Jones P Reid F. Altered mitochondrial function, mitochondrial DKDA and reduced metabolic flexibility in patients with diabetic nephropathy. EBioMedicine. (2015) 2:499–512. doi: 10.1016/j.ebiom.2015.04.002 26288815 PMC4534759

[34] Kim K Lee EY. Excessively enlarged mitochondria in the kiDKDeys of diabetic nephropathy. Antioxidants (Basel). (2021) 10:741. doi: 10.3390/antiox10050741 34067150 PMC8151708

[35] Barutta F Bruno G Grimaldi S Gruden G. Inflammation in diabetic nephropathy: moving toward clinical biomarkers and targets for treatment. Endocrine. (2015) 48:730–42. doi: 10.1007/s12020-014-0437-1 25273317

[36] Lim AK Ma FY Nikolic-Paterson DJ Kitching AR Thomas MC Tesch GH. Lymphocytes promote albuminuria, but not renal dysfunction or histological damage in a mouse model of diabetic renal injury. Diabetologia. (2010) 53:1772–82. doi: 10.1007/s00125-010-1757-1 20422398

[37] Chang TT Kuchroo VK Sharpe AH. Role of the B7-CD28/CTLA-4 pathway in autoimmune disease. Curr Dir Autoimmun. (2002) 5:113–30. doi: 10.1159/000060550 11826754

[38] Abais JM Zhang C Xia M Liu Q Gehr TW Boini KM. NADPH oxidase-mediated triggering of inflammasome activation in mouse podocytes and glomeruli during hyperhomocysteinemia. Antioxid Redox Signal. (2013) 18:1537–48. doi: 10.1089/ars.2012.4666 PMC3613176 23088210

